# Twin Screw Melt Granulation: A Single Step Approach for Developing Self-Emulsifying Drug Delivery System for Lipophilic Drugs

**DOI:** 10.3390/pharmaceutics15092267

**Published:** 2023-09-01

**Authors:** Dinesh Nyavanandi, Preethi Mandati, Sagar Narala, Abdullah Alzahrani, Praveen Kolimi, Sateesh Kumar Vemula, Michael A. Repka

**Affiliations:** 1 Department of Pharmaceutics and Drug Delivery, School of Pharmacy, The University of Mississippi, Oxford, MS 38677, USA; ndinesh624@gmail.com (D.N.); pmandati@go.olemiss.edu (P.M.); snarala@go.olemiss.edu (S.N.); aalzahra@go.olemiss.edu (A.A.); pkolimi@go.olemiss.edu (P.K.); svemula@olemiss.edu (S.K.V.); 2 Pii Center for Pharmaceutical Technology, The University of Mississippi, Oxford, MS 38677, USA

**Keywords:** self-emulsifying drug delivery system, liquid SEDDS, solid SEDDS, twin screw melt granulation, globule size, polydispersity index

## Abstract

The current research aims to improve the solubility of the poorly soluble drug, i.e., ibuprofen, by developing self-emulsifying drug delivery systems (SEDDS) utilizing a twin screw melt granulation (TSMG) approach. Gelucire^®^ 44/14, Gelucire^®^ 48/16, and Transcutol^®^ HP were screened as suitable excipients for developing the SEDDS formulations. Initially, liquid SEDDS (L-SEDDS) were developed with oil concentrations between 20–50% *w*/*w* and surfactant to co-surfactant ratios of 2:1, 4:1, 6:1. The stable formulations of L-SEDDS were transformed into solid SEDDS (S-SEDDS) using a suitable adsorbent carrier and compressed into tablets (T-SEDDS). The S-SEDDS has improved flow, drug release profiles, and permeability compared to pure drugs. The existence of the drug in an amorphous state was confirmed by differential scanning calorimetry (DSC) and powder X-ray diffraction analysis (PXRD). The formulations with 20% *w*/*w* and 30% *w*/*w* of oil concentration and a 4:1 ratio of surfactant to co-surfactant have resulted in a stable homogeneous emulsion with a globule size of 14.67 ± 0.23 nm and 18.54 ± 0.55 nm. The compressed tablets were found stable after six months of storage at accelerated and long-term conditions. This shows the suitability of the TSMG approach as a single-step continuous manufacturing process for developing S-SEDDS formulations.

## 1. Introduction

In today’s world, around 60–70% of the new chemical entities (NCEs) within the developmental pipeline are claimed to be poorly soluble, affecting oral bioavailability. Thus, the primary prerequisite of developmental scientists is to improve the solubility and bioavailability of biopharmaceutical classification system (BCS) class II and IV drug molecules [1,2,3,4,5]. Various strategies have been investigated to improve the solubility and oral bioavailability of drug substances with poor aqueous solubility. The investigated approaches include amorphous solid dispersions, complexation, cocrystals, co-amorphous systems, salt form, micronization, nanosizing, liposomes, and self-emulsifying drug delivery systems (SEDDS) [6,7,8]. Among various strategies, SEDDS approach was selected for further investigation. SEDDS are the isotropic mixtures of oil, surfactant, and co-surfactant/co-solvent, which results in the oil formation in a water type (o/w) of emulsion when brought in contact with aqueous media with slight agitation.

However, formulating L-SEDDS into soft gelatin capsules is time consuming, and expensive. In addition, the compatibility of L-SEDDS formulation with the soft gelatin capsule needs to be investigated [9,10,11]. The associated disadvantages of L-SEDDS have laid the path for developing solid SEDDS (S-SEDDS) formulations. Within S-SEDDS formulations, the liquid formulation is adsorbed onto a suitable solid carrier which can be further encapsulated or compressed into tablets. The development of S-SEDDS has improved the shelf life of the formulations, thereby overcoming the problems associated with L-SEDDS. To date, no formulations for the S-SEDDS are available in the market. Conventionally S-SEDDS are prepared by high-shear granulation, spray drying, and extrusion-spheronization techniques [12]. However, these manufacturing processes are considered as batch manufacturing, where the quantity of material being processed is limited depending on the size of the manufacturing equipment. In addition, these manufacturing processes need optimization of various process parameters, considered critical during scale-up or scale-down of the batch size, making them unsuitable for commercial scale. The pharmaceutical industries have huge developmental costs due to the significant disadvantages of poor drug solubility and underdeveloped manufacturing processes.

Both the industries and regulatory agencies such as Food & Drug Administration (FDA) are keenly looking for the establishment of a continuous manufacturing process where the quality of the product can be controlled and monitored by employing process analytical technology (PAT) tools which will, in turn, reduce the in-process and finished product testing making the product readily available in the market soon after manufacturing. In recent years twin screw granulation (TSG) has been most widely investigated as a single step continuous process over batch manufacturing. Further, the TSG process is categorized as twin-screw melt granulation (TSMG), twin-screw dry granulation (TSDG), and twin-screw wet granulation (TSWG) depending on the physical state of the binder. Along with TSG, other single step processes which have been investigated for developing lipid-based formulations include aqueous heat method, vapor deposition and electro spraying. These techniques are limited due to the challenges associated with large scale manufacturing [13,14,15,16,17].

The current investigation is focused on evaluating the suitability of the TSMG approach as a single-step continuous manufacturing process for developing S-SEDDS. Ibuprofen, a non-steroidal anti-inflammatory drug (NSAID), is a BCS class II molecule with a water solubility of 21 mg/L, a melting point of 75.0–77.0 °C, Log P of 3.8, pKa of 4.43, Log D of 1.68, and solubility of 25.16 μg/mL at pH 7.4 has been chosen as a model drug [18]. Various lipids such as Gelucire^®^ 44/14, Gelucire^®^ 48/16, Gelucire^®^ 50/13, Transcutol^®^ HP, and Maisine^®^ CC were screened for selection of suitable combinations of excipients for developing stable SEDDS formulations. The HLB of Gelucire^®^ 44/14 [melting point (MP): 42.5–47.5 °C], Gelucire^®^ 48/16 [MP: 46.0–50.0 °C], Gelucire^®^ 50/13 [MP: 46.0–51.0 °C], Maisine^®^ CC and Transcutol^®^ HP was found to be 11, 12, 11, 1 and 4 respectively. The Gelucire^®^ 44/14 existed as semi-solid block. Whereas, Gelucire^®^ 48/16, Gelucire^®^ 50/13 are available as pellets. The main purpose of choosing solid excipients is to favor the process of melt granulation and to result a final formulation in solid state which is suitable for compression. The suitable excipients for developing SEDDS were screened by conducting solubility and miscibility studies. Initially, L-SEDDS with varied concentrations of oil (20–50% *w*/*w*) and surfactant to co-surfactant ratios (2:1, 4:1, and 6:1) were developed by laboratory approach in scintillation vials. The advanced L-SEDDS formulations were characterized for globule size, emulsification time, robustness to dilution, and thermodynamic stability. The stable L-SEDDS formulations were developed into S-SEDDS using a twin screw melt granulation approach using a suitable solid adsorbent carrier and compressed into tablets and disintegrants. The formulations of S-SEDDS were characterized by various emulsification and tableting properties.

## 2. Materials and Methods

### 2.1. Materials

Ibuprofen (99.3% of purity, white crystalline powder) was purchased from Spectrum chemicals (New Brunswick, NJ, USA). All the lipid excipients Gelucire^®^ 44/14, Gelucire^®^ 48/16, Gelucire^®^ 50/13, Transcutol^®^ HP, and Maisine^®^ CC were kindly gifted by Gattefosse (Paramus, NJ, USA). The solid adsorbent carriers Neusilin^®^ US2 was gifted by Fuji chemical industries (Burlington, NJ, USA), Starch 1500^®^ (partially pregelatinized maize starch) was donated by Colorcon (Harleysville, PA), and Avicel PH^®^ 102 (microcrystalline cellulose) was donated by FMC Biopolymer (Philadelphia, PA, USA). All the disintegrants AC-DI-SOL^®^, Sodium starch glycolate, and Kollidon-CL^®^ were donated by FMC Biopolymer (Philadelphia, PA, USA), Roquette (Geneva, IL, USA), and BASF (Ludwigshafen, Germany), respectively. The Slide-A-Lyzer^TM^ mini dialysis device with a molecular weight of 10 kDa was procured from ThermoFisher Scientific (Hampton, NH, USA). All other chemicals and solvents utilized in the current investigation are of analytical grade.

### 2.2. Screening of Lipid Excipients

Screening of suitable excipients for developing SEDDS was performed using the previously reported method [19]. The solubility of ibuprofen was investigated in all the selected excipients Gelucire^®^ 44/14, Gelucire^®^ 48/16, Gelucire^®^ 50/13, Transcutol^®^ HP, and Maisine^®^ CC. The solubility was determined by melting 1.0 g of lipid in a scintillation vial at 50.0 ± 0.5 °C, to which weighed amount (25 mg) of the drug was added and mixed continuously for 10 min at 2000 rpm. After 10 min, the sample was observed for undissolved drug particles and drug precipitation upon cooling. The addition of the drug (25 mg) was continued until undissolved drug particles or precipitation was observed. The drug concentration until no precipitation was observed is noted as saturation solubility. The temperature was constant for all the excipients (solids and liquids). The maximum amount of drug dissolved in each excipient was reported (mg/g). All the samples were analyzed in triplicates (n = 3).

### 2.3. Development of L-SEDDS

Initially, formulations of L-SEDDS were developed using a laboratory melting and mixing approach in scintillation vials. The formulations were developed using varying oil concentrations ranging from 20–50% *w*/*w* with a different surfactant to co-solvent ratios of 2:1, 4:1, and 6:1. The surfactant concentrations and co-surfactant ranged from 33–68% *w*/*w* and 7–27% *w*/*w*, respectively. The drug load was maintained constant for all the prepared formulations. The amount of drug equivalent to 20% *w*/*w* of the total formulation weight (oil, surfactant, and co-surfactant) was added for all the formulations. The formulations were prepared by dissolving the weighed amount of drug in the surfactant, dispensing it into a scintillation vial, and adding co-surfactant and oil. The resultant mixture was mixed continuously for 10 min at 2000 rpm until a homogeneous isotropic mixture was obtained by maintaining the formulation at 50.0 ± 0.5 °C. The resulting formulations were stored at ambient room temperature until further characterization. All the formulations that are developed are shown in Table 1.

### 2.4. Miscibility of Excipients within the Formulations

Besides the solubility of the drug in excipients, miscibility also plays a vital role in developing a robust formulation that remains stable throughout its shelf-life. Therefore, immiscible formulations need not be considered for further characterization. The miscibility of all the developed formulations was characterized with and without the active substance. All the formulations of L-SEDDS were developed in a 3 mL scintillation vial in the presence and absence of the drug (Placebo) using the procedure described in Section 2.3. The formulations were left at ambient conditions for 24 h. The samples were visually inspected for any phase separation of excipients, exuding liquid, or drug precipitation from the formulations.

### 2.5. Construction of Ternary Phase Diagram

The stability of all the L-SEDDS formulations was assessed by adding 1 mL of the L-SEDDS formulation to a beaker containing 300 mL of distilled (DI) water subjected to continuous mixing at 100 rpm for 10 min. The samples were left at ambient room temperature for 48 h and observed for any change in the physical appearance of the solution, such as cloudiness, precipitation, and phase separation. Any formulation, if identified for any change in physical appearance, is considered unstable. All the formulations were investigated in triplicate to see the reproducibility of the observations. The ternary phase diagram was plotted to identify the self-emulsification region across the investigated concentrations using Tri plot v1-4 software (David Graham and Nicholas Midgley, Loughborough, Leicestershire, UK).

### 2.6. Characterization of L-SEDDS

#### 2.6.1. Self-Emulsification Time

The stable formulations were investigated to determine the self-emulsification time by adding 1 mL of L-SEDDS formulation to the dissolution vessel of 500 mL DI water using a type II (paddle) apparatus (SR8—Plus^TM^, Hanson Research, Chatsworth, CA, USA) at 50 rpm of speed by maintaining the media at 37 °C. The time for the incorporated formulation to disperse uniformly and result in a homogeneous emulsion was noted as self-emulsification time.

#### 2.6.2. Determination of Globule Size

The mean globule size and polydispersity index (PDI) were measured for all the stable formulations by photon correlation spectroscopy using Zetasizer Nano ZS Zen3600 (Malvern Instruments, Southborough, MA, USA) at 25 °C in a disposable clear cell. The globule size and PDI were measured using a helium-neon laser. The formulations were diluted 100 folds using double distilled water and filtered through a 0.45 μm nylon filter. All the samples were investigated in triplicate.

#### 2.6.3. Effect of pH on Stability of L-SEDDS

Lipid-based formulations maintain the drug in solubilized form for a prolonged period within the gastrointestinal tract. However, any change in the pH of the media might affect the stability of the formulation, thereby resulting in the precipitation of the drug. Therefore, the formulations of L-SEDDS were subjected to a series of dilutions (50, 100, and 1000 times) in enzyme-free gastric media (pH 1.2), enzyme-free intestinal media (pH 6.8 phosphate buffer), and water. The diluted samples were stored at ambient conditions for 24 h and inspected for any changes in the physical appearance, such as drug precipitation, phase separation, or change in solution clarity.

#### 2.6.4. Thermodynamic Stability and Centrifugal Stress Study

The stability of L-SEDDS formulations was evaluated by exposing the formulations to a cool heat cycle (45 °C and 4 °C) and freeze-thaw cycle (−21 °C and 25 °C) by storing the samples for 48 h at each temperature. The centrifugal stress was performed by diluting the formulations of L-SEDDS for 100 folds and subjecting them to centrifugation at 3500 rpm for 15 min (AccuSpin Micro 17, Fisher Scientific, Hampton, VA, USA). The samples were investigated for any phase separation of the formulation components and characterized for globule size, PDI, and emulsification time.

#### 2.6.5. Cloud Point Measurement

The stable formulations of L-SEDDS were evaluated for cloud point temperature. The temperature at which the formulation material tends to precipitate, resulting in turbidity or cloudiness indicating instability of the formulation, is called cloud point temperature. The cloud point temperature must be greater than 37 °C, more significant than the average body temperature. The cloud point temperature was measured by diluting the formulations for 100 folds and subjected to a linear temperature increase from 25 °C at a 2 °C/min rate using a water bath. The temperature at which precipitation or cloudiness was visually observed is noted as cloud point temperature. Similarly, the cloud point temperature of pure formulation components Gelucire^®^ 44/14, Gelucire^®^ 48/16, and Transcutol^®^ HP was determined for solutions with varying concentrations of excipients (1, 2, 3, 4, 5% *w*/*w*) in water. All the evaluations were performed in triplicate, and the average temperature (°C) ± standard deviation was reported.

### 2.7. Development of S-SEDDS

#### 2.7.1. Screening of Suitable Adsorbent Carriers

The stable formulations of L-SEDDS were further developed into S-SEDDS using a suitable adsorbent carrier. First, three excipients, Neusilin^®^ US2, Starch 1500^®^, and Avicel PH^®^ 102 (microcrystalline cellulose; MCC), were screened for suitability as a solid adsorbent carrier. Various properties of the carrier materials are described in Table 2. The screening was performed by taking 2.0 g of L-SEDDS (Placebo) formulation in a porcelain dish. Then, an increasing amount of the solid carrier was added and mixed thoroughly until a mixture with good flow properties was obtained. Next, the resulting free-flowing powder’s bulk density and tapped density were determined, and the flow properties were estimated using Carr’s index and Hausner’s ratio. Finally, the adsorption capacity was calculated for all the carrier materials using the equation:

$$Adsorption\;capacity=\frac{Weight\;of\;solid\;carrier}{weight\;of\;the\;formulation}$$

#### 2.7.2. Twin Screw Melt Granulation (TSMG)

The formulations of S-SEDDS were developed using an 11 mm hot melt extruder (Process11, Thermo Fisher Scientific, Waltham, MA, USA) by a twin screw melt granulation (TSMG) approach. Before preparing the physical mixtures, Gelucire^®^ 44/14, available as a solid block, was powdered by cryo-milling using dry ice and passed through the #30 ASTM sieve. All the materials (Gelucire^®^ 44/14, Gelucire^®^ 48/16, Transcutol^®^ HP) along with the drug and Neusilin^®^ US2 were dispensed and blended for 10 min at 20 rpm in a V-cone blender (Maxiblend, Globe Pharma, New Brunswick, NJ, USA) and collected into an airtight polybag until further processing. The physical mixtures of all the formulations were prepared for a batch size of 100 g. The process of TSMG was carried out at 80 °C (zone 3 to zone 8) barrel temperature using standard screw configuration (three mixing zones) with an identical screw speed of 50 rpm and feed rate of 3.0–3.5 g/min for all the formulations. The feed zone was maintained at 25 °C of temperature. The feed rate was calibrated to ensure the uniform flow of the material into the barrel. The granules were collected after the process torque was saturated, suggesting the uniform barrel fill volume. The process parameters were recorded, and the residence time of the material inside the barrel was measured by adding a few drops of food color (Green). The time taken for the colored material to exit the discharge point from the additional time was noted. The collected material was passed through the #25 ASTM sieve and packed in an airtight polybag for further characterization. A detailed description of formulation compositions investigated for S-SEDDS is shown in Table 3. The formulations of S-SEDDS were characterized for globule size, PDI, and emulsification time using the methods described to characterize L-SEDDS formulations in Section 2.6 respectively.

#### 2.7.3. Characterization of Granules

##### Flow Properties

The flow properties of the granules were determined by calculating Carr’s index (CI) and Hausner’s ratio using the following equations:

$$Carr’s\;index\;\left(CI\right)=\frac{Tapped\;density-Bulk\;density}{Tapped\;density}\times 100$$

$$Hausner’s\;ratio=\frac{Tapped\;density\;}{Bulk\;density}$$

Bulk density is the volume occupied by the weighed granules (10.0 g) when transferred into a measuring cylinder. The bulk density is calculated using the equation:

$$Bulk\;density=\frac{weight\;of\;material\;(g)}{volume\;(mL)}$$

Tapped density is the final volume occupied by the blend after tapping the measuring cylinder until no further reduction in volume is observed. The tapped density was calculated using the equation:

$$Tapped\;density=\frac{weight\;of\;material\;(g)}{Tapped\;volume\;(mL)}$$

##### Differential Scanning Calorimetry

The solid-state properties of the pure excipients, including the drug and the formulations of S-SEDDS, were characterized using Discovery DSC 25 (TA Instruments DSC, New Castle, DE, USA) mounted with an RCS90 cooling device. The instrument was calibrated for temperature and heat capacity using indium and sapphire standards. The sample weight equivalent to 5 mg was sealed in a Tzero aluminum pan enclosed with a Tzero aluminum lid with a hole in the center using a manual hand press. The thermal scans were studied from 25 °C to 90 °C with a linear temperature increase of 10 °C/min. An inert atmosphere was maintained using nitrogen with a 50 mL/min flow rate. The difference in the heat flow between the test and reference sample was plotted against the change in temperature using Trios software V4.0 (TA Instruments, New Castle, DE, USA).

##### Hot Stage Microscopy

The solvent capacity of the lipid mixtures to dissolve the crystalline drug was monitored using hot-stage microscopy (HSM). The HSM analysis was performed with polarized optical microscopy using an Olympus BX50 microscope mounted with a Metler Toledo FP-82 hot stage. The apparent solubility of the drug within the lipid excipients concerning temperature change was captured using Olympus DP73 digital camera. The nature of the drug within the samples was initially observed at ambient temperature (25 °C), and the apparent solubility within the molten lipid excipient was monitored at 50 °C. The physical mixtures of S-SEDDS formulations without solid adsorbent carrier Neusilin^®^ US2 were prepared and characterized for HSM analysis.

##### Powder X-ray Diffraction (PXRD)

The solid-state properties of the drug within the formulations of S-SEDDS were investigated using the Rigaku X-ray system (D/MAX-2500PC, Rigaku Corp, Tokyo, Japan) using Cu rays (λ = 1.54056 Å) with a current of 40 mA and voltage of 40 kV at ambient room temperature. All the scans were performed from 2–50° at a rate of 2°/min and width of 0.02°/s.

##### Fourier Transform Infrared Spectroscopy (FTIR)

The drug–excipients compatibility and formation of any interactions were investigated for the S-SEDDS preparations using Agilent Cary 660 FTIR spectrometer (Agilent Technologies, Santa Clara, CA, USA). The sample quantity of approximately 1 mg was placed on top of the diamond crystal and compressed using MIRacle high-pressure clamp. The samples were scanned 16 times with a 4 cm^−1^ resolution from 600 to 4000 cm^−1^. The instrument was mounted with attenuated total reflection (Pike Technologies, Madison, WI, USA) with an ingle bounce and a diamond-coated ZnSe internal reflection element.

##### Surface Morphology of Granules

The surface morphology of granules was captured using scanning electron microscopy (SEM, JSM-7200FLV, JOEL, Peabody, MA, USA) with a voltage of 5 kV. The sample was spread as a fine thin layer on top of a double adhesive tape adhered to the SEM stubs. Before capturing surface morphology, the samples were coated with platinum under an argon atmosphere using Denton Desk V TSC sputter coater (Denton Vacuum, Moorestown, NJ, USA).

##### Transmission Electron Microscopy (TEM)

The globules’ morphology and the S-SEDDS preparations’ average size were studied using TEM analysis. The sample was prepared by adding 1.0 g of formulation to 300 mL of distilled water and continuously mixed for 10 min at 200 rpm. After 10 min, the solution was filtered using a 0.45 μm nylon filter and characterized for surface morphology. The sample quantity of 20 μL was placed on a clean parafilm sheet on top of which a freshly glowed 200 mesh copper grid coated with thin carbon film was floated. The grids were removed after 30 s, and the excess sample was wiped off using Kimwipes. Before complete drying, the gird was placed on a drop of ultrapure water and removed immediately. The excess water was removed, and the grid was coated using a single drop of 1% uranyl acetate. The samples were visualized on a JEOL JEM-1400 Flash TEM, and the images were captured using Gatan One View digital camera.

##### In Vitro Dissolution Studies

The in vitro drug release profiles were investigated for S-SEDDS formulations and pure crystalline drugs in three different dissolution medias under non-sink conditions, i.e., water, 0.1N HCl (pH 1.2), and pH 7.2 phosphate buffer (USP dissolution media). The release studies used USP type II apparatus (SR8—Plus^TM^, Hanson Research) in 900 mL of media with 50 rpm paddle speed at 37 ± 0.5 °C of temperature. An aliquot of the sample (1 mL) was withdrawn at 5, 10, 15, 20, 30, 45, and 60 min. The samples were centrifuged for 10 min at 3000 rpm and filtered through a 0.45 μm nylon filter connected to the syringe. The drug content was measured using high-performance liquid chromatography (HPLC). The samples for dissolution were prepared by filling the granules of weight equivalent to 50 mg of ibuprofen into empty capsule shells of size 000.

The drug content was measured using Phenomenex Luna^®^ C18 reverse-phase column (5 μm, 100 Å, 250 × 4.6 mm). The mobile phase was prepared using water, acetonitrile, and chloroacetic acid in a ratio of 40:60:0.01 and sonicated for 30 min before usage. The samples were analyzed at a 2 mL/min flow rate with an injection volume of 10 μL. The UV—detector was set at 221 nm wavelength (Waters 2489 UV/detector). The results were analyzed using Empower 2 software. The calibration curve was plotted for the pure drug in water, 0.1N HCl, and pH 7.2 phosphate buffer with concentrations ranging from 1 to 100 μg/mL with a correlation coefficient (R^2^) of 0.998, 0.999, and 0.997, respectively. The limit of detection (LOD) and limit of quantification (LOQ) was found to be 0.3 and 1.0 μg/mL, respectively [20].

##### In Vitro Drug Diffusion

The diffusion behavior of S-SEDDS formulations was investigated using a Thermo Scientific^TM^ Slide-A-Lyzer^TM^ MINI Dialysis device with a 10 K molecular weight cutoff. S-SEDSS formulations and pure drug suspensions were prepared in distilled water at 2 mg/mL concentration. The suspension samples (0.3 mL) were pipetted into the donor chamber of 0.5 mL capacity and mounted on top of a 20 mL scintillation vial filled with pH 7.2 phosphate buffer, which serves as receiving media. The entire setup was kept for continuous mixing (200 rpm) by maintaining a constant temperature of 37 ± 0.5 °C throughout the study. An aliquot (1 mL) of the sample was collected at each time point (0.5, 1, 2, 4, 6, 8, 10, 12, 16, 20, 24 h) and replaced with the same quantity of receiving media. The drug content was measured using HPLC.

### 2.8. Compression of Tablets

The optimized formulation of S-SEDDS was further developed into tablets (T-SEDDS) by incorporating suitable disintegrants as extra granular material. Three disintegrants, namely AC-DI-SOL^®^, Sodium starch glycolate, and Kollidon-CL^®^ were screened at concentrations of 5 and 10% *w*/*w*. The granules of S-SEDDS and disintegrants were blended homogeneously using a V-blender for 10 min at 20 rpm. Then, the tablets were compressed using a single-punch manual compaction machine (Natoli, NP-RD10A) loaded with one set of oval-shaped punches (length × width: 19.0 × 11.0 mm). The compressed tablets were evaluated for globule size and PDI using the method described in Section 2.6.2 employed to characterize L-SEDDS formulations.

### 2.9. Characterization of Tablets

The compressed tablets of S-SEDDS formulations were evaluated for physicochemical characteristics such as weight variation, hardness, friability, disintegration, thickness, drug content, in vitro dissolution, globule size, and PDI.

#### 2.9.1. Weight Variation

Twenty tablets of each formulation were weighed individually, and the percent variation of each tablet from the average weight was calculated. All the tablets must be within ±5% of the average weight, and not more than two tablets can be outside the acceptable range.

#### 2.9.2. Dimensions

The thickness of the tablets was measured for ten tablets, and the average ± SD was reported. Uniform thickness with no variations shows signs of uniform weight, compression pressure, and hardness among the tablets. The thickness of tablets was measured using a vernier scale (VWR1, Radnor, PA, USA).

#### 2.9.3. Hardness

Ten formulation tablets were measured for the breaking force using a hardness tester (Schleuniger, Pharmatron, Westborough, MA, USA), and the mean hardness ± SD was reported.

#### 2.9.4. Friability

The friability of tablets was measured for ten tablets (greater than 6.50 g) using an FT2 friability apparatus (Schleuniger, Pharmatron). The group weight of ten tablets was noted, and the test was run for 4 min at 25 rpm. Following the test run, the samples were collected and dedusted, observed defects such as cracks, capping, and lamination, and the weight of tablets was recorded. The percentage friability was calculated using the following:

$$\%Friability=\frac{Intial\;weight-Fianl\;weight}{Initial\;weight}\times 100$$

#### 2.9.5. Disintegration

The disintegration of tablets plays a vital role in drug release profiles. The disintegration (Dr. Schleuniger Pharmatron, DT2-IS) test for compressed tablets was performed by placing six tablets into the basket tubes, suspended in 900 mL of water maintained at 37 °C. The time taken for all the tablets to disintegrate and pass through the mesh (#10 sieve) located at the bottom of the tubes is noted, and the average time ± SD was reported.

#### 2.9.6. Drug Content

Ten tablets of each formulation were pulverized into fine powder in a clean mortar, and the blend quantity equivalent to 10 mg of the drug was weighed and transferred into a volumetric flask (100 mL). The extraction solvent (as per the USP monograph) was prepared by dissolving 4.0 g of chloroacetic acid in 400 mL of water, and the pH was adjusted to 3.0 using ammonium hydroxide. Next, 600 mL of acetonitrile was mixed thoroughly with the above solution. The drug extraction process was done by sonicating the volumetric flask for 60 min, and centrifugation at 3000 rpm for 10 min. Next, the supernatant was collected by passing through a 0.45 μm filter and diluted suitably using water. Finally, the drug content was measured using HPLC.

#### 2.9.7. In Vitro Dissolution of Tablets

The compressed tablets were evaluated for drug release profiles in water, 0.1N HCl, and pH 7.2 phosphate buffer. In addition, the formulations were investigated using the method described above to characterize drug release profiles from the granules.

### 2.10. Stability Studies

The tablets of optimized formulation were loaded into a stability chamber (CARON 6030) at accelerated (40°C ± 2 °C/75% RH ± 5% RH) and long-term (25°C ± 2 °C/60% RH ± 5% RH) conditions for 06 months. The tablets were packed in an HDPE container with a 2.0 g of silica sachet placed inside the container. The samples were collected at 1, 3, and 06 months. In addition, the samples were evaluated for globule size, PDI, tablet hardness, friability, disintegration, content uniformity, in vitro dissolution, and DSC. The stability samples’ in vitro drug release profiles were compared with the initial release profiles (0 days) by calculating the similarity factor (f_2_).

$$f_{2}=50log\{[1+(\frac{1}{n})\sum_{t=1}^{n}\left(R_{t}-T_{t}{)}^{2}{]}^{-\frac{1}{2}}\times 100\right\}$$

where n is the number of sampling time points, R_t_ and T_t_ are the cumulative release rates of the reference and test samples. The similarity factor of ≥50 indicates closeness between the release profiles.

## 3. Results & Discussions

### 3.1. Screening of Lipid Excipients

**Figure 1:**
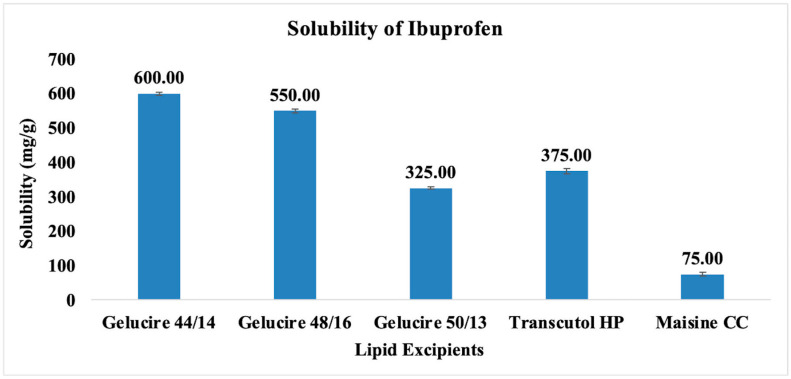
Solubility of the drug (ibuprofen) in various lipid excipients. The solubility is represented as the amount of drug dissolved in one gram of lipid.

Selecting suitable oil, surfactant, co-surfactant, or co-solvent is essential in developing SEDDS formulations. A single excipient might not be capable of dissolving the entire drug dose. However, employing a combination of compatible excipients can dissolve the entire dose of the drug and can maintain the drug in solubilized form without precipitation or recrystallization. The solubility of ibuprofen in all the investigated lipid excipients is shown in Figure 1. The order of solubility was found to be Gelucire^®^ 44/14 > Gelucire^®^ 48/16 > Transcutol^®^ HP > Gelucire^®^ 50/13 > Maisine^®^ CC. Among all the investigated excipients, Gelucire^®^ 44/14 has higher drug solubility of 600.0 mg/g of lipid, which can be attributed to its surfactant-like properties with good solubilizing potential. Therefore, Gelucire^®^ 44/14, a PEGylated lipophilic component is chosen as an oil phase for further investigations [21]. The solubility of the drug in Gelucire^®^ 48/16 was 550.0 mg/g of lipid. Gelucire^®^ 48/16 is a water-soluble non-ionic surfactant suitable for dissolving hydrophobic or lipophilic drug substances attributed to its higher HLB value of 16. It can form a micellar system when developed as a binary mixture in combination with active alone or in the presence of any co-surfactant or co-solvent. The surfactants with high HLB value are preferred for developing SEDDS formulations. With increasing HLB value, the material’s hydrophilic nature improves, resulting in rapid formation of o/w emulsion and rapid spreading of the formulation when brought in contact with the aqueous media. In addition, Gelucire^®^ 48/16 adsorbs on the surface of oil droplets due to its amphiphilic nature, thereby preventing the coalescence of oil droplets and improving their stability. Therefore, Gelucire^®^ 48/16 has been selected as a surfactant for developing SEDDS formulations. The solubility of the drug in Transcutol^®^ HP was found to be 375.0 mg/g of lipid. Transcutol^®^ HP serves as a powerful solubilizer and is utilized as a co-surfactant within the current investigation. Based on the solubilizing capacity of the excipients, Gelucire^®^ 44/14, Gelucire^®^ 48/16, and Transcutol^®^ HP were chosen as oil, surfactant, and co-surfactant for developing SEDDS formulations of ibuprofen.

### 3.2. Miscibility of Excipients within the Formulations

Along with solubility, the miscibility of formulation components also plays a vital role in developing stable formulations. All the formulations listed in Table 1 were prepared with (non-placebo) and without (placebo) addition of drug and evaluated for miscibility upon storage at ambient room temperature for 24 h. All the formulations were stable with no phase separation or leaching of liquid excipient upon storage. This shows the stability and miscibility of the formulation components within the investigated concentrations. In addition, upon the investigation of the non-placebo formulations, no observations of drug recrystallization or precipitation were noted. This shows the excipients’ solubilizing capacity and the maintenance of the drug in a dissolved state.

### 3.3. Construction of Ternary Phase Diagram

**Figure 2:**
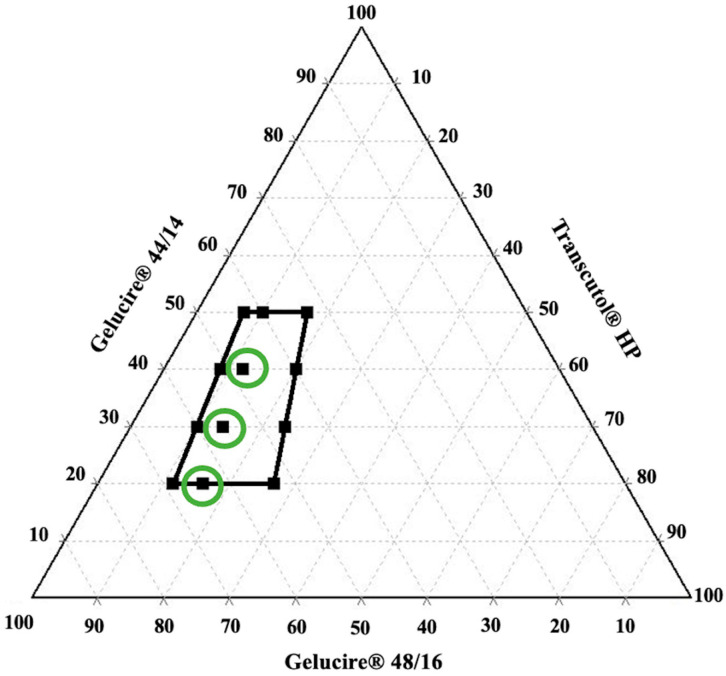
Ternary phase diagram representing emulsification region (black box) among the investigated concentrations. The green circled points represent the stable formulation after 48 h of storage at ambient temperature.

The formulations of L-SEDDS (F1–F12) were evaluated for stability upon storage at ambient temperature, and the emulsification region within the range of investigated concentrations is shown in Figure 2. All the formulations of L-SEDDS resulted in stable emulsions at 0 h of time point. However, the formulations dispersed rapidly in the bulk solution, which can be attributed to the high HLB value of the surfactant. After 48 h of storage, the stability of emulsions was not preserved for all the formulations. The formulations consisting of surfactant to co-surfactant (S/Co-s) ratio of 4:1 with 20, 30, and 40% *w*/*w* of oil phase (F2, F5, F8) have resulted in stable emulsions. Whereas the formulations consisting of S/Co-s ratios of 2:1 and 6:1 have resulted in poor stability with cloudiness or milky appearance and phase separation of the emulsions. The ratio of S/Co-s plays a vital role in developing stable formulations.

Furthermore, a relation exists between the concentration of surfactant and globule size. Increasing surfactant concentration results in reduced globule size attributed to the stabilization of oil droplets due to surfactant deposition at the interface of oil and the aqueous phase. Whereas, in a few cases, excess surfactant concentration beyond the beyond the optimum level results in larger globules attributing to greater hydrophilicity on the surface, resulting in water penetration into the globules resulting in the ejection of oil droplet into the aqueous phase [22,23,24]. Thus, an optimized surfactant concentration needs to be employed in developing stable formulations of SEDDS with smaller globules. Therefore, all the downstream characterization tests were performed for the stable formulations (F2, F5, F8). The physical appearance of all the formulations (F1–F12) before and after the storage is described in Supplementary Table S1.

### 3.4. Characterization of L-SEDDS

All the investigated formulations have resulted in homogeneous clear emulsions immediately when brought in contact with aqueous media. The rapid diffusion of the formulations into the bulk of the solution can be attributed to the nature of the formulation components with aqueous solubility and superior emulsification properties. Additionally, miscibility between the formulation components within the investigated concentrations has also contributed to forming a transparent, stable homogeneous emulsion. The emulsification time of the investigated L-SEDDS formulations was found to be ranging between 9.0–23.0 s. Increasing the oil concentration from 20% *w*/*w* to 40% *w*/*w* increased the emulsification time.

Similarly, the globule size of the formulations was increased with the increasing oil concentration, which aligns with the results published by Mercuri et al. (2011) [25]. The emulsions have resulted in the formulation of globules in the nanoscale range, due to which the physical appearance of the solutions was clear, with no observations of cloudiness or turbidity. The emulsions with particle size less than 30 nm result in the formation of clear transparent dispersions. In contrast, the emulsions with particle sizes ranging between 30–150 nm result in translucent dispersion, and the appearance of emulsions with particle sizes more significant than 150 nm exists as turbid or milky white [26]. The resulting emulsions’ PDI was less than 0.1 for formulations with 20 and 30% *w*/*w* of oil concentration and 0.13 for the formulation with 40% *w*/*w* of oil concentration indicating homogeneous dispersion [27]. Smaller globule size might result in greater oral absorption. Although a detailed mechanism pertaining to the relation between the globule size and absorption is not available, a few in vivo studies conducted by other researchers have shown greater absorption and bioavailability of formulations possessing smaller globule size. In research conducted by Smidt et al. (2004) [28], the penclomedine based emulsion with 160 nm globule size has resulted in greater oral bioavailability in rats when compared with the emulsion of 720 nm globule size. Still more research is warranted for in-depth understanding of the globule size effect on the mechanism of absorption and other pharmacokinetic parameters. The emulsification properties of the investigated formulations are shown in Table 4.

The cloud point temperature of the stable formulations was 68–72 °C, indicating the formulation’s stability when administered to the human patient population. The cloud point temperature has decreased with increasing oil concentration from 20–40% *w*/*w*. Upon investigation, the cloud point temperature of pure formulation components was found to be 72 °C, 69 °C, and 65 °C for Gelucire^®^ 44/14, Gelucire^®^ 48/16, and Transcutol^®^ HP, respectively. Interestingly, the cloud point temperature for all the investigated formulations ranges between 68.0 ± 5.0 °C, where 68 °C is the average cloud point temperature of all the pure formulation components (Gelucire^®^ 44/14, Gelucire^®^ 48/16, and Transcutol^®^ HP). The cloud point temperature of all the formulation components is shown in Supplementary Figure S1.

When exposed to different pH conditions, the stability of L-SEDDS formulations (F2, F5, F8) was evaluated in water, enzyme-free pH 1.2 simulated gastric fluid, and enzyme-free pH 6.8 simulated intestinal fluid. All the formulations under investigation remained stable when exposed to different pH environments except the formulation with 40% *w*/*w* of oil concentration (F8). The instability of the formulation, when diluted for 1000 folds, has resulted in phase separation of components after 24 h of storage at ambient conditions. The phase separation and instability of the formulation components can be attributed to the insufficient concentration of the surfactants, which might have been below the CMC when diluted for 1000 folds. The results of the formulations investigated for the robustness of dilution are shown in Supplementary Table S2.

The stability of formulations (F2, F5) when exposed to different conditions, heat cool, freeze-thaw cycles, and centrifugation stress were evaluated and characterized for globule size, PDI, and emulsification time. All the investigated formulations have remained stable after exposure to heat cool, freeze-thaw cycles, and centrifugal stress studies. The formulations were stable, with no phase separation or drug precipitation observations. The globule size of the formulations ranged between 13.0–17.0 nm with a PDI of less than or equal to 0.1, which aligns with the observations of initial samples. The emulsification time remained unchanged following post-exposure of the formulations to the extreme heat, cold, and centrifugal force conditions. The emulsification time was found to be ranging between 8.0–13.0 s. The detailed observations of thermodynamic stability investigated for stable formulations (F2, F5) are shown in Table 5. Thus, attributing to the stability of formulations with 20% *w*/*w* (F2) and 30% *w*/*w* (F5) of oil concentration with a surfactant to the co-surfactant ratio of 4:1 was considered for the development of S-SEDDS formulations.

### 3.5. Development of S-SEDDS

The stable formulations of L-SEDDS (F2, F5) were considered for further development into S-SEDDS using suitable solid adsorbent carriers by the TSMG approach.

#### 3.5.1. Screening of Suitable Carrier

Three solid adsorbent carriers, Neusilin^®^ US2, Starch 1500^®^, and Avicel PH^®^ 102 (microcrystalline cellulose; MCC), were screened for developing the S-SEDDS formulations. Neusilin^®^ US2 was identified as a suitable carrier among all the investigated materials based on the calculated adsorption capacity and flow properties. The adsorption capacity of Neusilin^®^ US2 was found to be 0.5. Starch and MCC adsorption capacities were 1.67 and 1.93 for both the formulations under investigation. The superior adsorption capacity of Neusilin^®^ US2 can be attributed to the greater surface area of 371.88 m^2^/g when compared with starch and MCC, with a surface area of 0.19 m^2^/g and 0.06 m^2^/g, respectively [29,30]. In addition, Neusilin^®^ US2 has exhibited superior flow properties compared to starch and MCC formulations. The superior flow properties of Neusilin^®^ US2 can be attributed to its spherical surface morphology. The flow properties for pure carriers are in the order of Neusilin^®^ US2 > Starch 1500 > MCC. Carr’s index and Hausner’s ratio of Neusilin^®^ US2 formulations are found to be 14.23 ± 0.12 (Good) and 1.00 ± 0.22 (Excellent), respectively. Based on the observations of superior adsorption capacity and flow properties, Neusilin^®^ US2 is selected as a suitable carrier for the formulation development of S-SEDDS. The adsorption capacity and flow properties of investigated solid adsorbent carriers are shown in Supplementary Table S3.

#### 3.5.2. Twin Screw Melt Granulation

The formulations of S-SEDDS (S1, S2) were granulated successfully using the TSMG approach. All the process parameters employed for TSMG are recorded in Supplementary Table S4. The granulation was carried at a temperature (80 °C) more significant than the melting point of the drug (78 °C) to dissolve it entirely in the lipid excipients. The feed zone was maintained at ambient temperature to prevent the melting and bridging of materials which affects the feeding process. The die plate was disconnected from the extruder barrel to collect the granules at the discharge point. The screw speed was optimized at 50 rpm to provide sufficient residence time for the processing materials. The molten lipid excipients get exposed to thermal and mechanical shear resulting in uniform mixing and nucleation of granules. The feed rate was optimized at 3.0–3.5 g/min to match the screw speed of the extrusion process. High feed rates of 5.0–6.0 g/min with a screw speed of 50 rpm have resulted in over-barrel fill levels with higher process torque. Whereas the feed rate of 1.5–2.0 g/min has resulted in low barrel fill levels with low process torque values resulting in the extrusion of a higher number of fines. The process torque was low, ranging between 10–12%, attributed to the lubrication property of the molten lipid components within the extruder barrel. A short residence time of 80–90 s was observed for both the granulated formulations indicating a shorter exposure time of the formulation components to the thermal stress. The process was allowed to stabilize for 5 min to achieve uniform barrel fill volume, and the granules collected initially were considered process waste. The process loss of materials during TSMG was negligible. At the end of the granulation process, the yield reconciliation was 99.0–99.5% for both the developed formulations, indicating no excess loss of materials during the process and making the process suitable formulations with low drug loadings or potent active substances. The collected granules were sifted through #25 ASTM mesh and packed in an airtight polybag until further characterization.

#### 3.5.3. Characterization of S-SEDDS Granules

The granules of S-SEDDS formulations were characterized for the globule size, PDI, emulsification time, and flow properties. The granules’ emulsification time is between 15–17 s, more significant than the emulsification time of the L-SEDDS formulations. The emulsification time of the L-SEDDS formulation was between 9.0–12.0 s. The delayed emulsification time can be attributed to the entrapment of the emulsifying agents within the porous network of the solid adsorbent carrier (Neusilin^®^ US2), which might have delayed its solubility and diffusion of the formulation components into the bulk solution. The obtained emulsion was slightly turbid compared with the emulsions of L-SEDDS, attributed to Neusilin^®^ US2 particles, which remain insoluble when brought in contact with the media. The globule size of the granules ranged between 14.0–19.0 nm, with a PDI value of less than 0.1, indicating a homogeneous distribution of the globules with no significant effect of the solid carrier on the globule size and PDI. The flow behavior of the formulations plays an essential role in developing robust solid dosage forms. The formulations of S-SEDDS have resulted in a superior flow behavior where Carr’s index and Hausner’s ratio ranged between 7.0–9.0 and 1.04–1.07, respectively. The superior flow behavior of the granules can be attributed to spherical nature and higher adsorption capacity of Neusilin US2^®^. The characterization results of S-SEDDS formulations are shown in Table 6.

#### 3.5.4. Differential Scanning Calorimetry

**Figure 3:**
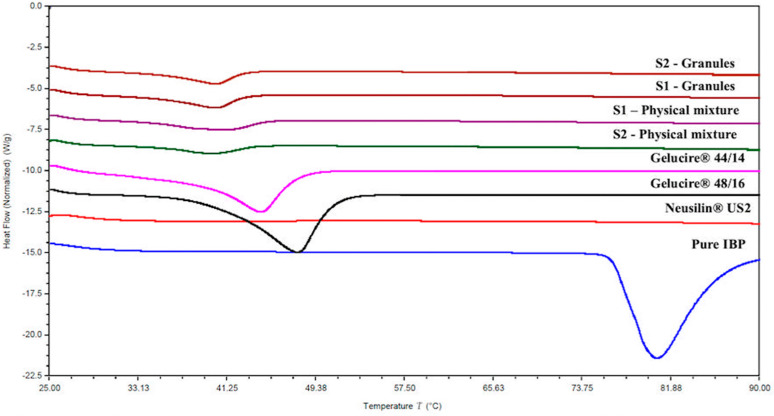
Thermal properties of pure drug, physical mixtures, pure excipients, and granules of S1 and S2 formulations.

The solid-state properties of the drug within the granules of S-SEDDS (S1, S2), physical mixtures, pure excipients, and the pure drug were characterized using DSC. The thermal scans of all the investigated formulations are represented in Figure 3. The pure drug resulted in an endothermic peak at 78 °C attributing to its melting point. The thermal scan of Neusilin^®^ US2 has resulted in a straight line indicating the amorphous nature of the solid carrier. The lipid excipients Gelucire^®^ 44/14 and Gelucire^®^ 48/16 have resulted in an endothermic peak at 48 °C and 44 °C, indicating the existence of lipid excipients in crystalline form. The thermal scans of the physical mixture and granules (S1, S2) have resulted in an endothermic melting peak of lipid excipients. In contrast, no melting peak of the drug was observed in both the thermal scans of physical mixtures and granules. The absence of the drug melting peak within the physical mixture can be attributed to the dissolution of the drug within the molten lipid material. However, the physical nature of the drug within the granules needs to be confirmed using hot-stage microscopy and PXRD analysis. The endothermic melting peaks of the pure formulation components are in line with previously reported literature [31,32,33,34].

#### 3.5.5. Hot-Stage Microscopy (HSM)

**Figure 4:**
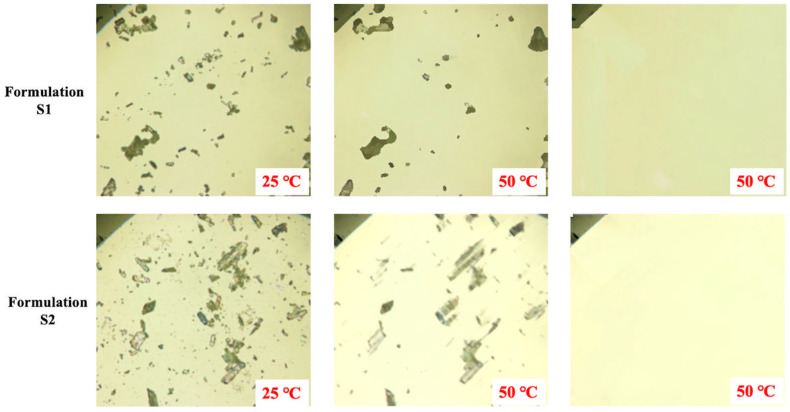
Hot-stage microscopy representing the solubilizing capacity of the lipid excipients. S1 and S2 represents the granule formulations.

The visual images depicting the apparent solubility of the drug within the lipid excipients for the temperature change are captured in Figure 4. The microscopic images of the formulations captured at room temperature show the drug’s existence in crystalline form. After heating the samples to 50 °C, which is above the lipid excipients’ melting point, the drug’s dissolution within the molten lipid was observed. Over time, no drug crystals were visible, indicating the solubilizing capacity of the ternary lipid mixtures that have dissolved the crystalline drug. The absence of an endothermic melting peak of the drug within the physical mixture in DSC can be attributed to the solubilizing capacity of the molten lipid mixture, which has dissolved the drug with increasing temperature. Thus, HSM has played an essential role in determining the physical state of the drug substance within the formulations and solvent capacity of the selected composition.

#### 3.5.6. Powder X-ray Diffraction Analysis (PXRD)

The physical state of the drug within the granules of S-SEDDS formulations (S1, S2) was evaluated using PXRD analysis. The diffractogram of pure ibuprofen has resulted in characteristic peaks at 6°, 16.5°, 19.5°, and 22°, respectively, indicating the crystalline nature of the drug. The solid adsorbent carrier Neusilin^®^ US2 has resulted in the absence of characteristic peaks indicating its amorphous nature. The diffractograms of lipid excipients Gelucire^®^ 44/14 and Gelucire^®^ 48/16 have resulted in characteristic peaks at two theta values of 19.1°, 23.1°, and 19.08°, 23.36° respectively. The characterization of granules has resulted in no characteristic peaks of the drug, confirming the transformation of the drug from its crystalline to amorphous nature.

**Figure 5:**
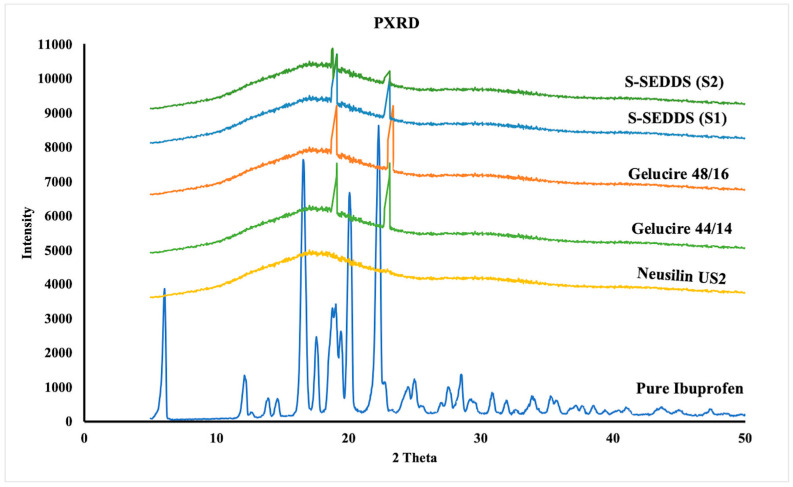
PXRD diffractograms of pure drug, Neusilin^®^ US2, Gelucire^®^ 44/14, Gelucire^®^ 48/16, and granules of S-SEDDS formulations (S1 & S2).

In contrast, the characteristic peaks of lipid excipients (Gelucire^®^ 44/14 and Gelucire^®^ 48/16) were observed in the diffractograms of the S-SEDDS formulation indicating the existence of lipids in crystalline form. All the observed characteristic peaks align with the literature [35,36,37]. Thus, PXRD analysis confirms the existence of the drug in amorphous form within the investigated formulations of S-SEDDS. The XRD diffractogram of all the investigated formulations is shown in Figure 5.

#### 3.5.7. Fourier Transform Infrared Spectroscopy

**Figure 6:**
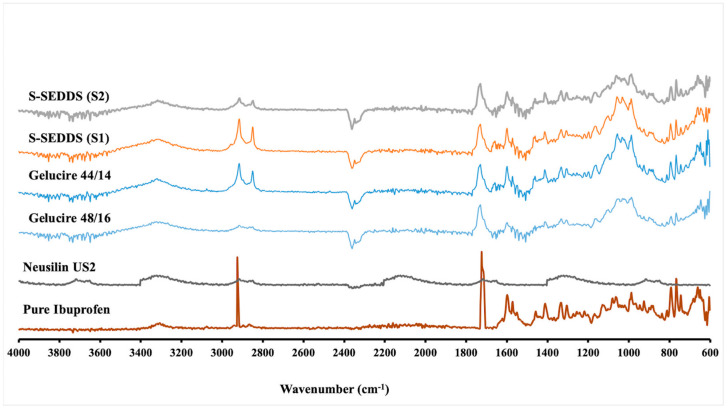
FTIR spectra of pure drug, Neusilin^®^ US2, Gelucire^®^ 44/14, Gelucire^®^ 48/16, and granules of S-SEDDS formulations (S1 & S2) (X-axis: wavenumber; Y-axis: absorbance).

The compatibility of the drug–excipient, and formation of any interactions between the formulation components was characterized using FTIR spectral analysis. The spectra of pure ibuprofen have shown the characteristic peaks at 668 cm^−1^ (C-H stretching), 779 cm^−1^ (CH_2_ rocking vibration), 1231 cm^−1^ (C-C stretching), 1721 cm^−1^ (C=O stretching vibrations), and 2920 (OH stretching). The spectra of Neusilin^®^ US2 have resulted in characteristic peaks at 3466 and 994 cm^−1^. Whereas characterization of pure lipid components (Gelucire^®^ 44/14 and Gelucire^®^ 48/16) has shown the characteristic peaks at 2885, 1736, 1467, 1341, 1103, 959, 842 cm^−1^ for Gelucire^®^ 48/16, and the characteristic peaks for Gelucire^®^ 44/14 were observed at 2929, 2856 cm^−1^ (C-H stretching), 1730 cm^−1^ (C=O stretching), and 1117 (C-O stretching). The characterization of FTIR spectra for the granules of S-SEDDS has resulted in all the characteristic peaks with no shifting or broadening, indicating no formation of interactions and compatibility between the formulation components [35,36,37]. The FTIR spectral analysis investigated for all the advanced S-SEDDS formulations is shown in Figure 6.

#### 3.5.8. Scanning Electron Microscopy

**Figure 7:**
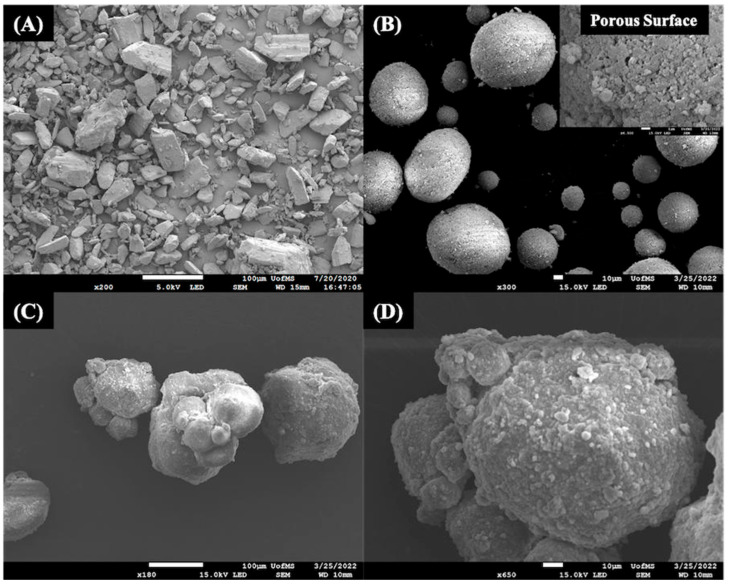
Scanning electron microscopy analysis for the (**A**) pure active substance, Ibuprofen (×200 magnification) (**B**) pure solid adsorbent carrier Neusilin^®^ US2 (×300 magnification) (**C**) granules of S-SEDDS; S1 (×180 magnification) (**D**) granules of S-SEDDS; S2 (×650 magnification).

The surface morphology of the S-SEDDS granules for the investigated formulations (S1, S2) was studied using SEM analysis, and the captured surface morphology of the granules is shown in Figure 7. The surface morphology of the active substance (Figure 7A) was found to have particles of irregular geometry with needle-like structures and a flat surface. The flat surface of the particles tends to have greater friction with the contact parts of the manufacturing instruments and results in poor flow behavior. The surface morphology of the solid adsorbent carrier Neusilin^®^ US2 (Figure 7B) was found to have spherical geometry with a porous surface. The molten formulation components (ibuprofen, Gelucire^®^ 44/14, Gelucire^®^ 48/16, and Transcutol^®^ HP) get adsorbed into the porous network of the Neuslin carrier. As a result, they remain stable without any phase separation and recrystallization of the drug throughout its shelf life. The surface morphology of the S-SEDDS granules (Figure 7C,D) was found to be waxy, attributing to the nature of the lipid; however, the spherical geometry of the solid adsorbent carrier was preserved, indicating superior flow behavior of the formulations. The surface morphology of the granules has no signs of drug crystals, indicating the presence of the drug in amorphous nature dissolved within the lipid excipients. The SEM analysis observations align with the results of DSC and PXRD studies. SEM analysis has served as a secondary confirmatory tool for determining the physical state of the active substance within the granules of the S-SEDDS formulations.

#### 3.5.9. Transmission Electron Microscopy

**Figure 8:**
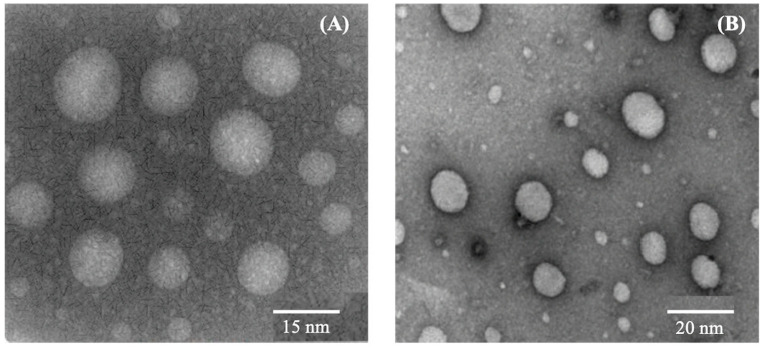
TEM images of S-SEDDS formulations (S1, S2). (**A**) Formulation S1 (**B**) Formulation S2.

The morphology of globules formed in the resulting emulsions of S-SEDDS formulations (S1, S2) is shown in Figure 8. The globules of both the investigated formulations were found to be spherical with no agglomeration. The particles’ mean size was 15 nm (S1) and 20 nm (S2) for formulations with 20% *w*/*w* and 30% *w*/*w* of oil concentration which is in line with the particle size of the L-SEDDS formulations. The globule size of the L-SEDDS formulations was 12.30 ± 0.16 nm (F2) and 15.54 ± 0.24 nm (F5), respectively.

#### 3.5.10. In Vitro Dissolution Studies

**Figure 9:**
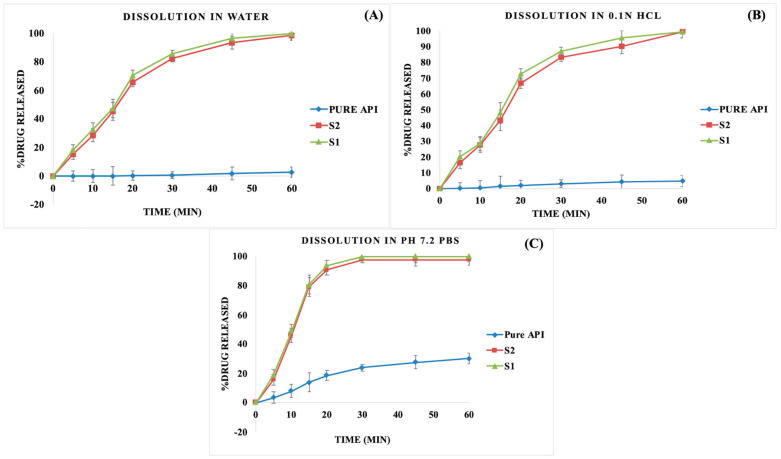
In vitro drug release profiles of S - SEDDS granules in (**A**) water, (**B**) 0.1N HCl, and (**C**) pH 7.2 phosphate buffer solution (PBS).

The in vitro drug release profiles of S-SEDDS formulations and pure drugs were investigated in water, 0.1N HCl, and pH 7.2 phosphate buffer solution (PBS). The investigated formulations of S-SEDDS have resulted in similar drug release profiles in all the investigated dissolution media with a similarity factor (f_2_) greater than 90. The dissolution of the pure drug has resulted in incomplete release profiles in water and 0.1N HCl, attributed to the crystalline poor soluble nature of the drug. The dissolution of the pure drug was 2.68% and 4.84% in water and 0.1N HCl at 60 min. Ibuprofen, being strongly acidic, results in poor solubility in an acidic environment and exhibits pH-dependent solubility. Ibuprofen has a pKa of 4.43, which results in more excellent solubility at a pH greater than its pKa. The dissolution of the pure drug in alkaline media (pH 7.2 PBS) at a pH greater than its pKa has resulted in improved drug release profiles. The dissolution of the pure drug in pH 7.2 PBS has resulted in 30.11% of drug release in 60 min. The formulations of S-SEDDS (S1, S2) have resulted in rapid drug release profiles in all the investigated media. A greater than 80% drug release was achieved in all the investigated dissolution media within 30 min. The drug release was faster in pH 7.2 PBS, which can be attributed to the more excellent solubility of the drug in an alkaline environment compared with other dissolution media. The dissolution profiles of all the investigated formulations for the granules of S-SEDDS formulations are shown in Figure 9.

#### 3.5.11. In Vitro drug Diffusion Studies

**Figure 10:**
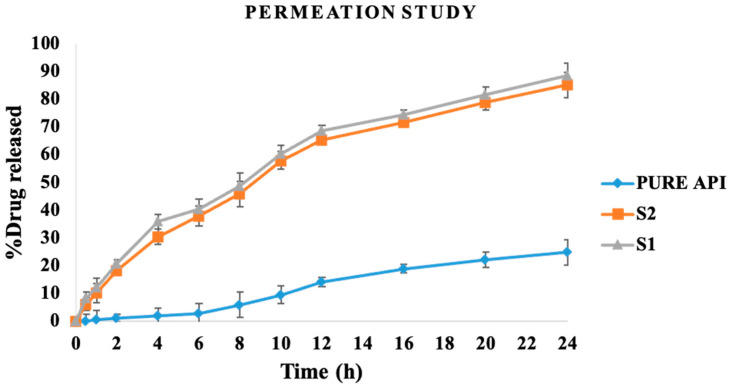
In vitro diffusion studies of S-SEDDS formulations (S1, S2) in comparison with pure drug.

The diffusion behavior of the drug within the formulations of S-SEDDS was evaluated using a dialysis membrane with a 10 kDa molecular weight cutoff. The emulsions of S-SEDDS formulations have exhibited improved drug permeability compared with the suspension of pure drugs. The formulations of S-SEDDS have resulted in 88.46% (S1) and 85.22% (S2) of drug permeation in 24 h. The suspension of the pure drug has resulted in 24.79% of drug permeation, which can be attributed to its poor crystalline solubility. The greater diffusion of the drug for formulation with 20% *w*/*w* of oil concentration (S1) can be attributed to the smaller size of globules compared with the formulation consisting of 30% *w*/*w* of oil concentration (S2). This shows the suitability of developing SEDDS in achieving improved solubility and permeability of poorly aqueous soluble drugs where the stability of formulations can be preserved by developing S-SEDDS. The in vitro diffusion of investigated formulations for 24 h is shown in Figure 10.

#### 3.5.12. Compression and Characterization of Tablets

The optimized formulations of S-SEDDS (S1, S2) were further developed into tablets (T1, T2) using suitable disintegrants to allow the disintegration of the tablet matrix and for rapid emulsification and dissolution profiles when brought in contact with the media. Three different disintegrants, namely AC-DI-SOL (Croscarmellose sodium), Sodium starch glycolate, and Kollidon—CL, were investigated at 5.0 and 10.0% *w*/*w* of concentrations. The tablets were compressed for a target weight of 395 mg (5% *w*/*w* of disintegrant) and 415 mg (10% *w*/*w* of disintegrant) to accommodate a dose equivalent to 50 mg of ibuprofen. A detailed description of all the tablet properties for the investigated formulations is shown in Supplementary Table S5. The tablets of both formulations were compressed for a hardness of 8.0–10.0 kp. The disintegrant concentration at 5.0% *w*/*w* has resulted in slower disintegration of the tablet matrix, resulting in delayed drug release profiles. The disintegration time for the formulations with 5.0% *w*/*w* of disintegrant was greater than 250 s. The disintegration order of the formulations with 5.0% *w*/*w* of disintegrant was sodium starch glycolate < croscarmellose sodium < Kollidon—CL. Increasing the disintegrant concentration to 10.0% *w*/*w* has reduced the formulations’ disintegration time with croscarmellose sodium and Kollidon CL. At the same time, the disintegration time of the formulation with 10.0% *w*/*w* of sodium starch glycolate has resulted in increased disintegration time from 250 s to 465 s attributing to the gelling properties of the disintegrant when incorporated at higher concentrations [37]. The formulations with 10.0% *w*/*w* of croscarmellose sodium have resulted in rapid disintegration of tablets in less than 50 s for both the investigated formulations. Increasing disintegrant concentration from 5.0 to 10.0% *w*/*w* has increased compressional force from 1.5 kN to 1.7 kN to achieve a target hardness of 8.0–10.0 kP, which shows a drop in compressibility properties of formulation mixture with increasing concentration of disintegrant. The use of any additional lubricants, such as magnesium stearate, to favor the tableting process was avoided since it delays the wetting and disintegration activity of the tablets attributing to its hydrophobic nature [37]. The weight of the tablets was found to be within ±5% of the target hardness. The friability of tablets for all the investigated formulations was less than 0.5% which is acceptable. An increased friability of tablets was observed with increasing concentration disintegrant, which is in line with the poor compressibility of the formulations. Thus, the formulations with 10.0% *w*/*w* of croscarmellose sodium compressed at 1.7 kN of compression force were optimized for further characterization.

The size of the resulting globules for the compressed tablet formulation was found to be 15.45 ± 0.23 nm (T1) and 18.78 ± 0.21 nm (T2) with a PDI value of less than 0.1, indicating homogeneous dispersion. The size of globules is in line with that of the granules (S1, S2) and L-SEDDS (F2, F5) formulations. This shows the negligible effect of the downstream processing on the quality of the formulations. The drug content ranged between 99.0–101.0%, indicating no loss and uniform distribution of active substances throughout the manufacturing process. The results of globule size, PDI, and content uniformity of the tablet formulations are shown in Table 7. The in vitro drug release profiles (Supplementary Figure S2) of compressed tablets (T1, T2) in water, 0.1N HCl, and pH 7.2 PBS were found to be in line with the release profiles of the S-SEDDS granules (S1, S2). A slower drug release profile was observed during the initial 05 min of time point, attributing to the disintegration of the tablets. The calculated similarity factor between the formulations of S-SEDDS granules (S1, S2) and tablets (T1, T2) was greater than 90, indicating similarity between the release profiles. Overall, the process of transforming L-SEDDS formulations into S-SEDDS granules and T-SEDDS has resulted in a decreased drug load from 20.0% *w*/*w* to 12.0% *w*/*w*. However, the quality attributes of the formulation were preserved resulting in successful development of T-SEDDS for which stability needs to be further evaluated.

#### 3.5.13. Stability Studies

The optimized formulations of SEDDS tablets (T1, T2) were evaluated for their stability upon storage at accelerated (40 °C ± 2 °C/75% RH ± 5% RH) and long-term (25 °C ± 2 °C/60% RH ± 5% RH) conditions for 06 months. The formulations were sampled at 01, 03, and 06 months of time points and characterized for solid-state properties of the drug, in vitro dissolution profiles, hardness, disintegration, friability, content uniformity, globule size, and PDI. The physicochemical properties, globule size, PDI, and drug content of tablets upon storage are captured in Supplementary Table S6. The hardness, friability, and disintegration times of tablets (T1, T2) were acceptable and aligned with the initial results (0 days). The hardness of tablets was found to range between the established limits of 8.0–10.0 kP, and the friability is less than 0.6% for both the investigated formulations (T1, T2). The disintegration of tablets was found to be between 40–60 s resulting in the rapid release of the drug from the tablet matrix. The stability and drug content were preserved even after storage at accelerated conditions for 06 months. The drug content of tablets was found to be 98.0–102.0%. The globules within the resulting emulsion are comparable with the globule size of L-SEDDS, granules of S-SEDDS, and initial tablets (0 days). The PDI of all the investigating formulations was less than 0.3, which is acceptable. The thermograms of the stability samples characterized using DSC have resulted in the existence of the drug in the amorphous state with no endothermic melting peak of the drug. The DSC thermal scans of the stability samples are shown in Supplementary Figure S3. The in vitro drug release profiles investigated for the stability samples in water, 0.1N HCl, and pH 7.2 PBS have resulted in similar drug release profiles, which align with the initial analysis results (0 days). The calculated similarity factor (f_2_) was greater than 90, indicating similarity between the release profiles. The drug release profiles of all the investigated stability samples are shown in Supplementary Figure S4.

## 4. Conclusions

The formulations of L-SEDSS were developed successfully using suitable lipid excipients Gelucire^®^ 44/14, Gelucire^®^ 48/16, and Transcutol^®^ HP by employing a laboratory melt mixing approach. The suitable concentration of oil and ratio of surfactant to co-surfactant was optimized by characterizing the formulations of L-SEDDS for various properties such as emulsification time, robustness to pH, thermodynamic stability, centrifugal stress, cloud point, globule size, and PDI. The stable formulations of L-SEDDS were developed into S-SEDDS granules by employing a suitable solid adsorbent carrier with superior adsorption capacity and flow properties. The formulation of S-SEDDS granules was developed using the TSMG approach. Upon transformation, the properties of L-SEDDS were preserved within the formulations of S-SEDDS. The optimized formulations of S-SEDDS were compressed into tablets using a suitable disintegrant. The compressed tablets have improved dissolution profiles and permeability compared with the pure drug. Upon storage at the long-term and accelerated conditions for six months, the stability of formulations was preserved and resulted in similar properties compared with the initial samples of L-SEDDS and the granules of S-SEDDS. Thus, the TSMG can be employed as an alternative single-step continuous manufacturing process for developing the SEDDS formulations for improving the solubility and permeability of poorly aqueous soluble drug substances.

## Figures and Tables

**Table 1:** A detailed description of all the formulation compositions developed for L-SEDDS.

Category	Excipient *	F1	F2	F3	F4	F5	F6	F7	F8	F9	F10	F11	F12
Oil	Gelucire^®^ 44/14	20.00	20.00	20.00	30.00	30.00	30.00	40.00	40.00	40.00	50.00	50.00	50.00
Surfactant	Gelucire^®^ 48/16	53.33	64.00	68.57	46.67	56.00	60.00	40.00	48.00	51.43	33.33	40.00	42.86
Co-solvent	Transcutol^®^	26.67	16.00	11.43	23.33	14.00	10.00	20.00	12.00	8.57	16.67	10.00	7.14
Total (%)		100.00	100.00	100.00	100.00	100.00	100.00	100.00	100.00	100.00	100.00	100.00	100.00
Ratio	^S/Co-S	2:1	4:1	6:1	2:1	4:1	6:1	2:1	4:1	6:1	2:1	4:1	6:1

A drug equivalent to 20% *w*/*w* of the formulation weight was added for all the formulations. * All the amounts were expressed in% *w*/*w* for all the formulations. ^S/Co-S—Surfactant to co-surfactant ratio.

**Table 2:** Various properties of carrier materials.

Properties	Avicel^®^ PH 102	Neusilin^®^ US2	Starch^®^ 1500
Loss on drying (%)	3.0–5.0	5.0	8.5
Bulk density (g/mL)	0.28–0.33	0.13–0.18	0.64
pH	5.5–7.0	6.0–8.0	5.0–7.0
Mean particle size (microns)	97	106	150
Molecular weight (g/mol)	370.35	143.37	NA
Surface area (m/g)	0.06	371.88	0.19
Melting Point/Glass Transition (°C)	247–250	NA	54–55 (Tg)
Degradation temperature (°C)	318–350	NA	250

NA: Not available. Tg: Glass transition temperature.

**Table 3:** Detailed composition of S-SEDDS formulations. Formulations S1 and S2 represent F2 and F5 formulations of L-SEDDS.

Material *	S1	S2
Gelucire^®^ 44/14	13.33	20.00
Gelucire^®^ 48/16	42.67	37.33
Transcutol^®^ HP	10.67	9.33
Neusilin^®^ US2	33.33	33.33
Total	100.00	100.00

* Each formulation has a drug load of 13.33% *w*/*w* which is equivalent to 20.0% *w*/*w* in L-SEDDS formulations.

**Table 4:** Emulsification time, globule size, PDI and cloud point of stable L-SEDDS formulations.

Formulation	Emulsification Time (s)	Globule Size (nm)	PDI	Cloud Point (°C)
F2	9.98 ± 2.23	12.30 ± 0.16	0.09 ± 0.02	72.00 ± 1.00
F5	12.11 ± 3.67	15.54 ± 0.24	0.06 ± 0.01	71.00 ± 1.00
F8	23.15 ± 2.77	18.33 ± 0.45	0.13 ± 0.02	68.00 ± 1.00

**Table 5:** Thermodynamic stability L-SEDDS formulations.

Formulation	Cycle	Size (nm)	PDI	Emulsification Time (s)
F2	Heat Cool	13.01 ± 0.11	0.10 ± 0.05	10.45 ± 1.23
	Freeze-Thaw	13.12 ± 0.21	0.09 ± 0.03	9.32 ± 1.66
	Centrifugation	13.98 ± 0.33	0.07 ± 0.02	8.01 ± 1.45
F5	Heat Cool	16.45 ± 0.07	0.06 ± 0.01	13.15 ± 2.43
	Freeze-Thaw	16.51 ± 0.11	0.09 ± 0.04	12.67 ± 1.31
	Centrifugation	15.33 ± 0.34	0.10 ± 0.01	0.10 ± 0.01

**Table 6:** Characterization of the granules of S-SEDDS formulations.

Formulation	Emulsification Time (s)	Globule Size (nm)	PDI	Carr’s Index	Hausner’s Ratio
S1	15.98 ± 1.23	14.67 ± 0.23	0.10 ± 0.02	7.45 ± 1.23	1.04 ± 0.02
S2	17.13 ± 2.45	18.54 ± 0.55	0.08 ± 0.01	8.67 ± 2.04	1.07 ± 0.04

**Table 7:** Globule size, PDI, and drug content of compressed tablet formulations.

Formulation	Size (nm)	PDI	Drug Content (%)
T1	15.45 ± 0.23	0.09 ± 0.02	99.45 ± 1.23
T2	18.78 ± 0.21	0.08 ± 0.04	101.67 ± 1.49

## Data Availability

The data presented in this study are available within the article and Supplementary Material.

## Supplementary Materials

The following supporting information can be downloaded at: https://www.mdpi.com/article/10.3390/pharmaceutics15092267/s1. Table S1. A detailed description of the L-SEDDS formulations physical appearance and stability at 0 h and 48 h of storage; Table S2. Stability of formulations when exposed to different pH conditions and dilutions; Table S3. Screening of suitable solid adsorbent carrier; Table S4. Process parameters of the TSMG process employed for developing S-SEDDS formulations; Table S5. Various physicochemical properties of compressed tablets (T1, T2); Table S6. Physiochemical characterization of SEDDS tablets upon storage at the long term and accelerated stability conditions for 06 months; Figure S1. Cloud point temperature of pure formulation excipients (Gelucire^®^ 44/14, Gelucire^®^ 48/16, and Transcutol^®^ HP) with concentrations ranging from 1–5 %w/w in water; Figure S2. In vitro drug release profiles of SEDDS tablets in (A) water, (B) 0.1N HCl, and (C) pH 7.2 PBS; Figure S3. Thermal characterization of SEDDS tablets after storage at the long term and accelerated conditions for 06 months; Figure S4. In vitro dissolution profiles of SEDDS tablets upon storage at the long term and accelerated conditions for 06 months.
