# Supplementary material for: Twin Screw Melt Granulation: A Single Step Approach for Developing Self-Emulsifying Drug Delivery System for Lipophilic Drugs
Source: Pharmaceutics. 2023 Sep 1;15(9):2267. doi: 10.3390/pharmaceutics15092267 (PMC10534719; doi:10.3390/pharmaceutics15092267)
Supplement: Supplementary file 1 [file pharmaceutics-15-02267-s001.zip › pharmaceutics-2581226-supplementary.pdf]

Supplementary Table S1. A detailed description of the L-SEDDS formulations physical appearance and stability at 0 h and 48 h of storage

**Formulation**	**Oil (%w/w)**	**S/co-s**	**Initial appearance (0 h)**	**After 48 h**	**Stability**
F1	20	2:1	Clear	Turbid	Unstable
F2	20	4:1	Clear	Clear	Stable
F3	20	6:1	Clear	Turbid	Unstable
F4	30	2:1	Clear	Turbid	Unstable
F5	30	4:1	Clear	Clear	Stable
F6	30	6:1	Clear	Turbid	Unstable
F7	40	2:1	Clear	Turbid	Unstable
F8	40	4:1	Clear	Clear	Stable
F9	40	6:1	Clear	Turbid	Unstable
F10	50	2:1	Clear	Phase separation	Unstable
F11	50	4:1	Clear	Phase separation	Unstable
F12	50	6:1	Clear	Phase separation	Unstable

Supplementary Table S2. Stability of formulations when exposed to different pH conditions and dilutions.

Formulation	pH 1.2			pH 6.8			Water		
	**50**	**100**	**1000**	**50**	**100**	**1000**	**50**	**100**	**1000**
F2	√	√	√	√	√	√	√	√	√
F5	√	√	√	√	√	√	√	√	√
F8	√	√	X	√	√	X	√	√	X

√ - indicates stable formulation

X – indicates unstable formulations

Supplementary Table S3. Screening of suitable solid adsorbent carrier

Formulation	Solid Carrier	Adsorption Capacity (%w/w)	Carr's Index (C.I)	Hausner's ratio
S1 (F2)	Neusilin® US2	0.50	14.23 ± 0.12 (Good)	1.00 ± 0.22 (Excellent)
	Starch 1500	1.67	20.13 ± 0.65 (Passable)	1.27 ± 0.24 (Passable)
	MCC PH 102	1.93	26.41 ± 0.13 (Poor)	1.35 ± 0.11 (Poor)
S2 (F5)	Neusilin® US2	0.50	14.65 ± 0.22 (Good)	0.98 ± 0.19 (Excellent)
	Starch 1500	1.67	21.11 ± 0.15 (Passable)	1.28 ± 0.08 (Passable)
	MCC PH 102	1.93	27.33 ± 0.27 (Poor)	1.36 ± 0.21 (Poor)

Supplementary Table S4. Process parameters of the TSMG process employed for developing S-SEDDS formulations

**Process Parameters**	**S1**	**S2**
Zone - 2 (°C)	25	25
Zone 3 - 8 (°C)	80	80
Die (°C)	25	25
Screw (rpm)	50	50
Feed rate (g/min)	3.0 - 3.5	3.0 - 3.5
Torque (%)	10 - 12	10 - 12
Residence time (Sec)	80 - 90	80 - 90

Supplementary Table S5. Various physicochemical properties of compressed tablets (T1, T2)

Formulation	Disintegrant	Concentration (%w/w)	Compressional Force (kN)	Hardness (kP)	Disintegration Time (Sec)	Weight variation (mg)	Friability (%)	Thickness (mm)
**T1 (S1)**	AC-DI-SOL	5.00	1.5 kN	9.45 ± 0.65	300.45 ± 15. 23	396.34 ± 2.45	0.28	3.45 ± 0.34
	Sodium starch glycolate	5.00	1.5 kN	9.66 ± 0.31	250.21 ± 23.44	394.33 ± 1.99	0.31	3.38 ± 0.24
	Kollidon -CL	5.00	1.5 kN	8.79 ± 0.11	475.33 ± 17.45	395.33 ± 3.45	0.29	3.25 ± 0.33
**T1 (S1)**	AC-DI-SOL	10.00	1.7 kN	9.26 ± 0.12	45.18 ± 10.45	415.67 ± 2.78	0.41	4.53 ± 0.45
	Sodium starch glycolate	10.00	1.7 kN	9.32 ± 0.33	465.34 ± 24.11	414.45 ± 3.67	0.44	4.50 ± 0.31
	Kollidon -CL	10.00	1.7 kN	9.54 ± 0.41	180.31 ± 33.61	415.21 ± 3.11	0.51	4.48 ± 0.22
**T2 (S2)**	AC-DI-SOL	5.00	1.5 kN	9.21 ± 0.11	290.21 ± 22.11	395.23 ± 1.44	0.26	3.38 ± 0.11
	Sodium starch glycolate	5.00	1.5 kN	8.99 ± 0.21	267.32 ± 11.21	396.33 ± 2.45	0.32	3.23 ± 0.35
	Kollidon -CL	5.00	1.5 kN	9.65 ± 0.47	483.34 ± 9.67	395.31 ± 2.11	0.30	3.40 ± 0.45
**T2 (S2)**	AC-DI-SOL	10.00	1.7 kN	9.33 ± 0.61	47.45 ± 16.43	416.77 ± 3.11	0.45	4.52 ± 0.33
	Sodium starch glycolate	10.00	1.7 kN	8.99 ± 0.11	475.55 ± 19.33	415.45 ± 2.66	0.46	4.45 ± 0.23
	Kollidon -CL	10.00	1.7 kN	9.11 ± 0.23	200.45 ± 21.41	414.33 ± 3.45	0.43	4.50 ± 0.11

Supplementary Table S6. Physiochemical characterization of SEDDS tablets upon storage at the long term and accelerated stability conditions for 06 months

**Storage (25/60)**	**Formulation**	**Tablet Hardness**	**Tablet Friability**	**Disintegration Time (Sec)**	**Drug Content (%)**	**Particle Size (nm)**	**PDI**	**f2**
1 M	T1	9.45 ± 0.32	0.45	40.45 ± 10.23	98.45 ± 2.67	15.67 ± 0.67	0.11 ± 0.02	98
	T2	8.89 ± 0.45	0.48	42.33 ± 9.45	99.78 ± 1.89	17.45 ± 0.33	0.13 ± 0.03	97
3 M	T1	9.34 ± 0.21	0.43	50.45 ± 12.31	101.45 ± 0.89	16.47 ± 0.31	0.09 ± 0.01	97
	T2	9.11 ± 0.45	0.40	49.67 ± 10.11	99.71 ± 2.76	18.45 ± 0.63	0.11 ± 0.04	95
6 M	T1	9.55 ± 0.23	0.51	45.21 ± 19.67	98.89 ± 1.41	15.89 ± 0.77	0.13 ± 0.06	98
	T2	9.65 ± 0.43	0.49	48.61 ± 21.56	99.67 ± 2.11	18.43 ± 0.39	0.16 ± 0.02	96

**Storage (40/75)**	**Formulation**	**Tablet Hardness**	**Tablet Friability**	**Disintegration Time (Sec)**	**Drug Content (%)**	**Particle Size (nm)**	**PDI**	**f2**
1 M	T1	9.32 ± 0.45	0.43	45.55 ± 9.23	101.33 ± 1.30	16.47 ± 0.37	0.09 ± 0.01	97
	T2	9.89 ± 0.33	0.41	41.43 ± 8.45	99.70 ± 2.80	17.40 ± 0.45	0.10 ± 0.04	96
3 M	T1	8.34 ± 0.20	0.55	51.35 ± 11.31	99.42 ± 0.50	15.40 ± 0.35	0.07 ± 0.02	95
	T2	9.23 ± 0.11	0.57	50.67 ± 10.15	100.71 ± 1.76	19.45 ± 0.60	0.12 ± 0.03	97
6 M	T1	8.45 ± 0.13	0.49	48.21 ± 20.67	101.89 ± 2.41	15.50 ± 0.55	0.09 ± 0.05	96
	T2	8.45 ± 0.23	0.47	47.50 ± 20.45	98.67 ± 1.09	18.45 ± 0.89	0.13 ± 0.03	95

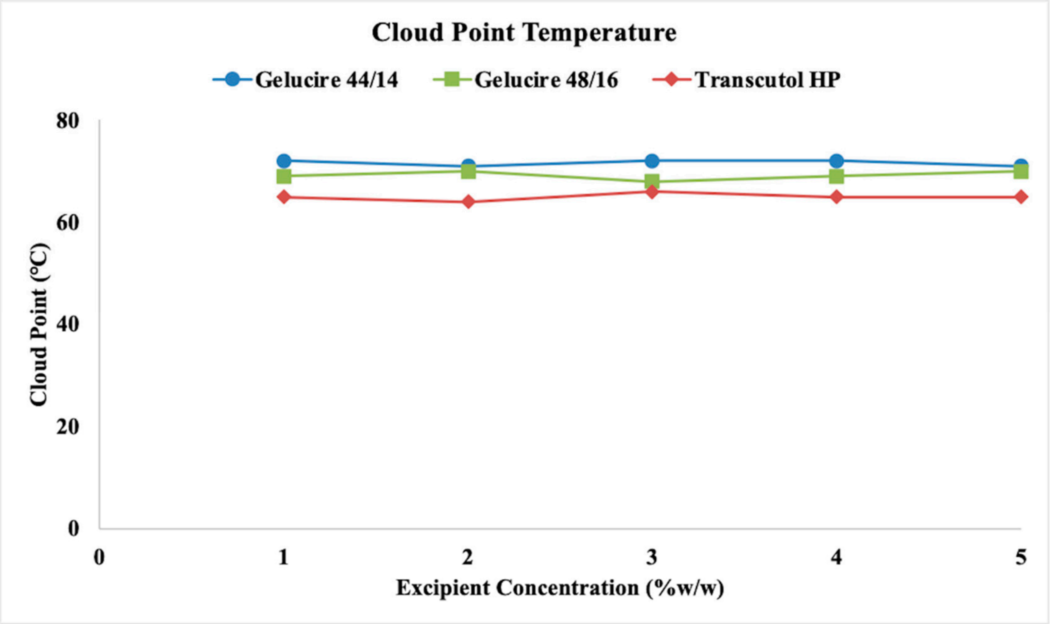

Supplementary Figure S1. Cloud point temperature of pure formulation excipients (Gelucire® 44/14, Gelucire® 48/16, and Transcutol® HP) with concentrations ranging from 1 – 5 %w/w in water.

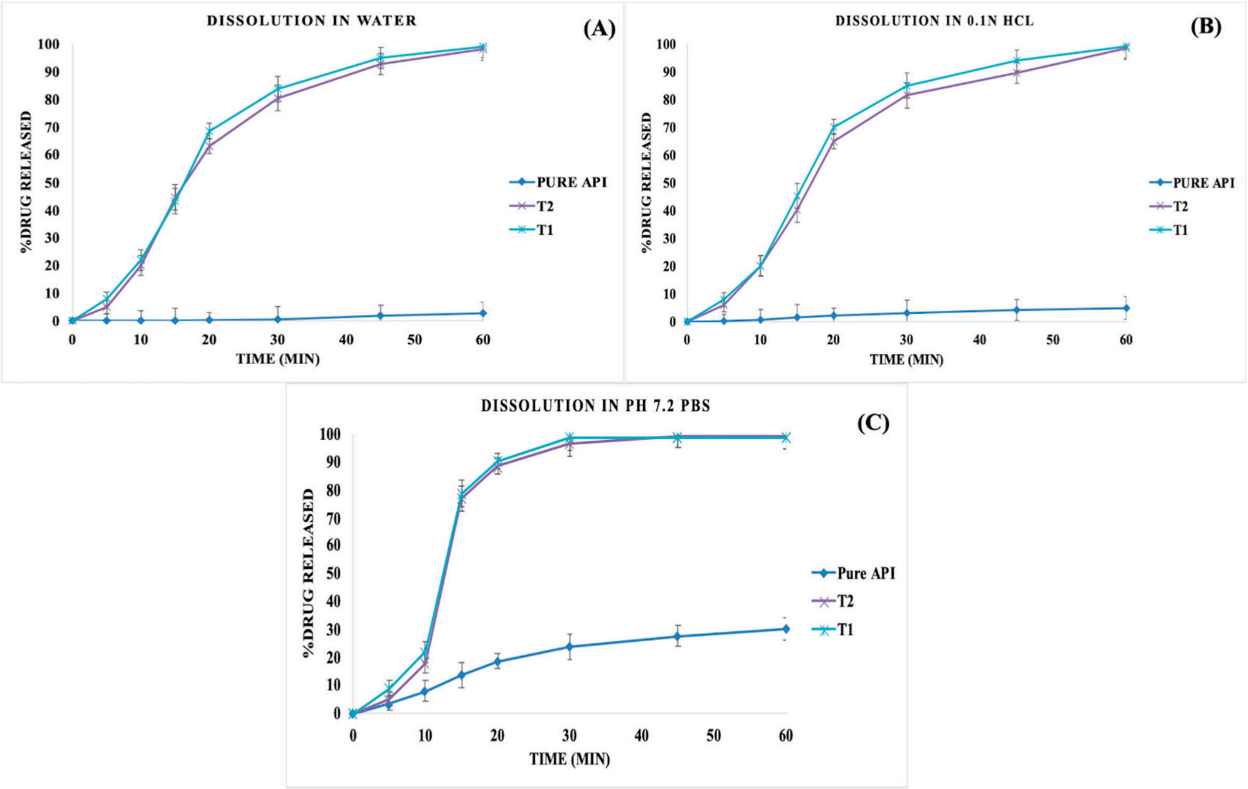

Supplementary Figure S2. *In vitro* drug release profiles of SEDDS tablets in (A) water, (B) 0.1N HCl, and (C) pH 7.2 PBS.

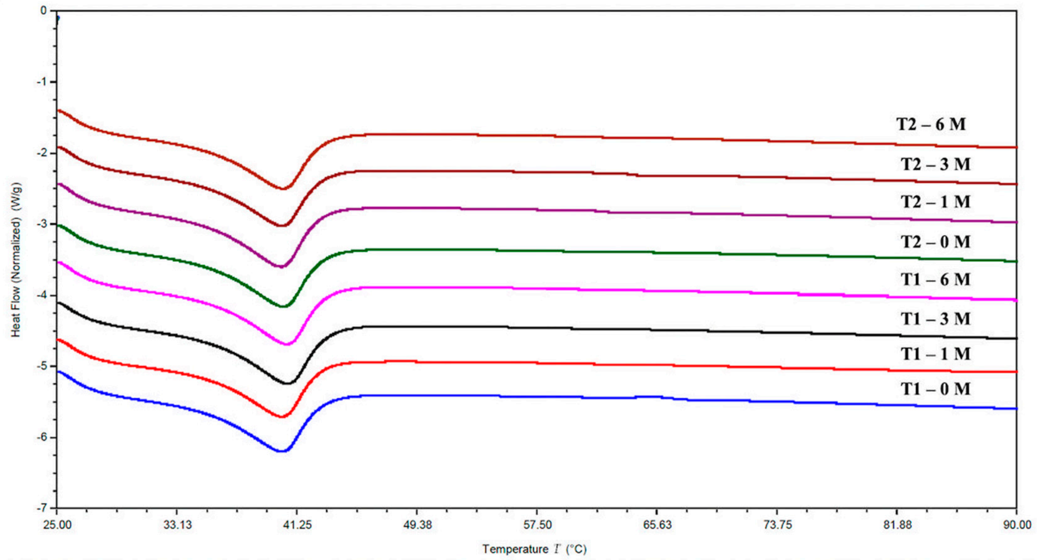

Supplementary Figure S3. Thermal characterization of SEDDS tablets after storage at the long term and accelerated conditions for 06 months

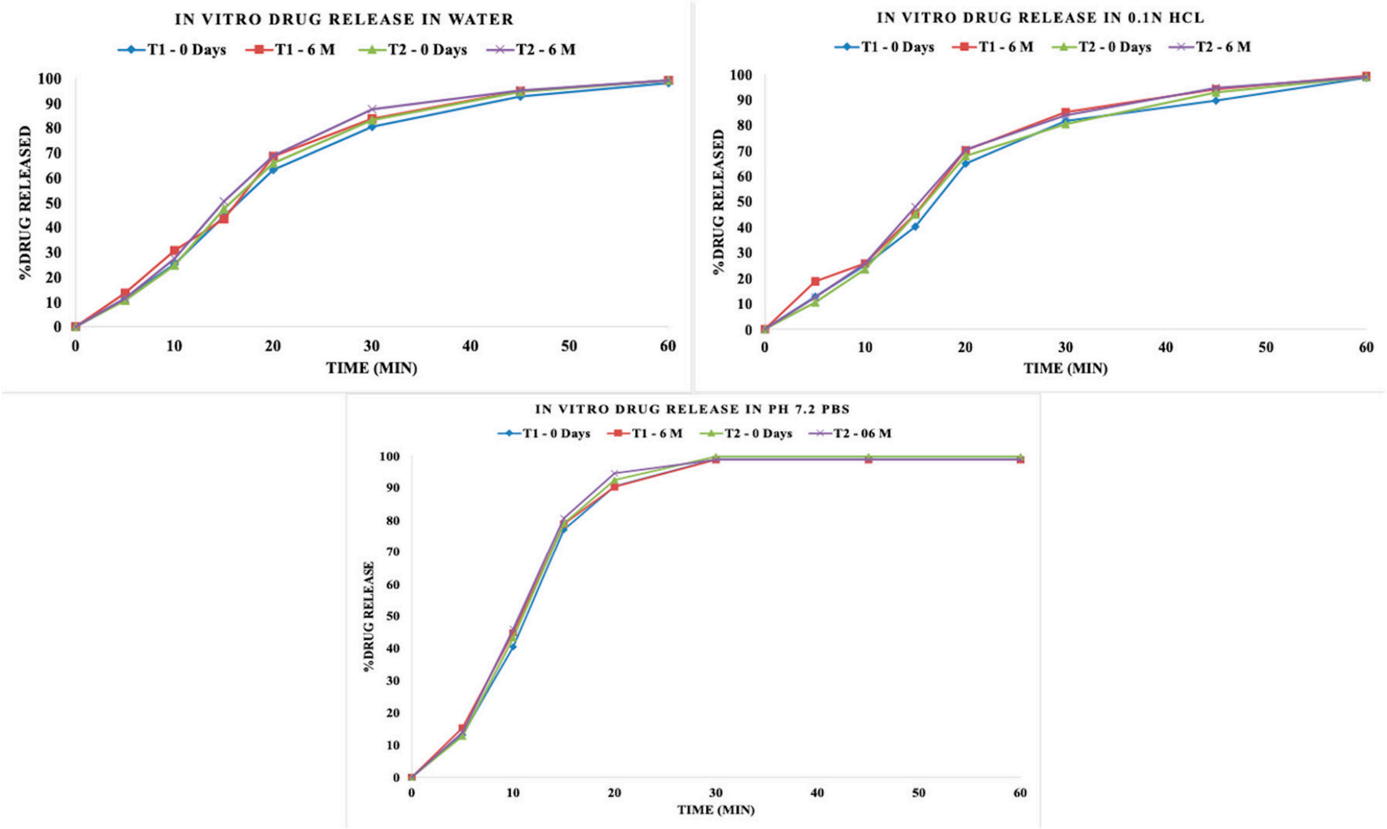

Supplementary Figure S4. *In vitro* dissolution profiles of SEDDS tablets upon storage at the long term and accelerated conditions for 06 months.
